# Genome Wide Analysis of Drug-Induced Torsades de Pointes: Lack of Common Variants with Large Effect Sizes

**DOI:** 10.1371/journal.pone.0078511

**Published:** 2013-11-06

**Authors:** Elijah R. Behr, Marylyn D. Ritchie, Toshihiro Tanaka, Stefan Kääb, Dana C. Crawford, Paola Nicoletti, Aris Floratos, Moritz F. Sinner, Prince J. Kannankeril, Arthur A. M. Wilde, Connie R. Bezzina, Eric Schulze-Bahr, Sven Zumhagen, Pascale Guicheney, Nanette H. Bishopric, Vanessa Marshall, Saad Shakir, Chrysoula Dalageorgou, Steve Bevan, Yalda Jamshidi, Rachel Bastiaenen, Robert J. Myerburg, Jean-Jacques Schott, A. John Camm, Gerhard Steinbeck, Kris Norris, Russ B. Altman, Nicholas P. Tatonetti, Steve Jeffery, Michiaki Kubo, Yusuke Nakamura, Yufeng Shen, Alfred L. George, Dan M. Roden

**Affiliations:** 1 Cardiovascular Sciences and Genetics Research Centers, St George’s University of London, London, United Kingdom; 2 Departments of Medicine, Molecular Physiology and Biophysics, Pediatrics, and Pharmacology, Vanderbilt University School of Medicine, Nashville, Tennessee, United States of America; 3 Pennsylvania State University, Eberly College of Science, The Huck Institutes of the Life Sciences, University Park, Pennsylvania, United States of America; 4 Tokyo Medical and Dental University, Bunkyo-ku, Tokyo, Japan; 5 Department of Medicine I, University Hospital Munich, Ludwig-Maximilians-University Munich, Munich, Germany; 6 Department of Biomedical Informatics, Columbia University, New York, New York, United States of America; 7 Heart Failure Research Center, Department of Clinical and Experimental Cardiology, Academic Medical Center, University of Amsterdam, Amsterdam, The Netherlands; 8 Institute for Genetics of Heart Diseases, Department of Cardiovascular Medicine, University Hospital Münster; 9 Institut National de la Santé et de la Recherche Médicale, UMRS 956, University Pierre et Marie Curie, Univ Paris 06, Paris, France; 10 Department of Medicine (Cardiology), University of Miami Miller School of Medicine, Miami, Florida, United States of America; 11 Drug Safety Research Unit, Southampton, United Kingdom; 12 Institut National de la Santé et de la Recherche Médicale, UMR1087, CNRS UMR 6291, Université de Nantes and CHU Nantes, Nantes, France; 13 Center of Cardiology at Hospital of Starnberg, Starnberg, Germany; 14 Department of Bioengineering, Stanford University, Palo Alto, California, United States of America; 15 Department of Biomedical Informatics, Columbia University, New York, New York, United States of America; 16 University of Chicago, Chicago, Illinois, United States of America; 17 RIKEN Center for Genomic Medicine, Yokohama, Japan; 18 Deutsches Zentrum für Herz-Kreislauf-Forschung e.V., partner site Munich Heart Alliance, Munich, Germany; 19 IZKF of the University of Münster, Münster, Germany; 20 Department of Physiology, University of Miami Miller School of Medicine, Miami, Florida, United States of America; 21 Department of Molecular and Cellular Pharmacology and Hussman Institute of Human Genomics, University of Miami Miller School of Medicine, Miami, Florida, United States of America; Sanjay Gandhi Medical Institute, India

## Abstract

Marked prolongation of the QT interval on the electrocardiogram associated with the polymorphic ventricular tachycardia Torsades de Pointes is a serious adverse event during treatment with antiarrhythmic drugs and other culprit medications, and is a common cause for drug relabeling and withdrawal. Although clinical risk factors have been identified, the syndrome remains unpredictable in an individual patient. Here we used genome-wide association analysis to search for common predisposing genetic variants. Cases of drug-induced Torsades de Pointes (diTdP), treatment tolerant controls, and general population controls were ascertained across multiple sites using common definitions, and genotyped on the Illumina 610k or 1M-Duo BeadChips. Principal Components Analysis was used to select 216 Northwestern European diTdP cases and 771 ancestry-matched controls, including treatment-tolerant and general population subjects. With these sample sizes, there is 80% power to detect a variant at genome-wide significance with minor allele frequency of 10% and conferring an odds ratio of ≥2.7. Tests of association were carried out for each single nucleotide polymorphism (SNP) by logistic regression adjusting for gender and population structure. No SNP reached genome wide-significance; the variant with the lowest P value was rs2276314, a non-synonymous coding variant in *C18orf21* (p  =  3×10^−7^, odds ratio = 2, 95% confidence intervals: 1.5–2.6). The haplotype formed by rs2276314 and a second SNP, rs767531, was significantly more frequent in controls than cases (p  =  3×10^−9^). Expanding the number of controls and a gene-based analysis did not yield significant associations. This study argues that common genomic variants do not contribute importantly to risk for drug-induced Torsades de Pointes across multiple drugs.

## Introduction

Marked prolongation of the QT interval on the electrocardiogram can occur in an unpredictable fashion during treatment with both QT interval-prolonging antiarrhythmic drugs as well as with drugs prescribed for non-cardiovascular indications [1], [2]. Such QT interval prolongation, in turn, is associated with a morphologically-distinctive and potentially fatal polymorphic ventricular tachycardia termed Torsades de Pointes (TdP). This severe adverse event (SAE), drug-induced Torsades de Pointes (diTdP), occurs in 1–5% of patients exposed to QT-prolonging antiarrhythmics (most often prescribed for atrial fibrillation, AF), and is much less common in patients treated with “non-cardiovascular” QT culprit drugs. Clinical risk factors have been identified and these include female gender, hypokalemia, bradycardia, and recent conversion from AF to normal rhythm. Basic electrophysiologic studies have demonstrated that inhibition of a major repolarizing potassium current, I_Kr_, is the common mechanism across multiple drugs and drug classes [1]–[3]. In addition, for specific drugs, inhibition of metabolism or elimination leading to accumulation of high drug concentrations in plasma has been implicated. This SAE has been a leading cause for drug withdrawal and relabeling, and evaluation of the potential that a new drug entity will carry a risk of diTdP has become a standard component of new drug evaluation [4].

A number of lines of evidence suggest that genomic variants may contribute to variable susceptibility to diTdP. The syndrome phenocopies many of the clinical features of the congenital long QT syndrome, a disease caused by mutations in genes encoding ion channels or their function modifying subunits [3], [5], and case series have identified mutations in these genes in a minority of patients with diTdP but normal QT intervals at baseline [6]–[8]; this is thought to represent incomplete penetrance of the congenital syndrome. Further evidence for a genetic contribution to diTdP is provided by a report that QT interval responses to challenge with the antiarrhythmic quinidine were greater among first-degree relatives of patients with diTdP than among controls [9]. Finally, the duration of the QT interval itself is known to be heritable [10]-[13], and multiple genomic loci have been highly significantly and reproducibly associated with this variability [14], [15].

The genome-wide-association study (GWAS) paradigm has previously been used to identify strong signals for susceptibility to SAEs using small numbers (<100) cases [16]–[18]. We present here the results of a GWAS to test hypothesis that common genomic variation contributes to risk for diTdP. Cases were accrued by two multicenter collaborations and compared to drug-challenged controls manifesting no major QT interval prolongation as defined below and to population controls. In this analysis, we find no highly statistically significant association between common single nucleotide polymorphisms and risk for diTdP in a Northwestern European population.

## Methods

### Cases

Cases of diTdP reported here were ascertained using the common definition below by two groups: the Trans-Atlantic Alliance Against Sudden Death supported by the Fondation Leducq and the Drug-induced Arrhythmia Risk Evaluation (DARE) study [5]. The Leducq set was collected by six North American and European University Medical centers: Vanderbilt, Miami, Munster, Amsterdam, Nantes, and Munich. The DARE study is a systematic national study of cases of British drug-induced arrhythmia identified prospectively over an initial 5 year period (2003-8).

A common case definition of diTdP was utilized: documented polymorphic ventricular tachycardia; typical pause-dependent initiation; association with exposure to a known or likely culprit drug with resolution of TdP and at least partial resolution of QT prolongation with removal of drug exposure. QT interval prolongation was also required; this was most often documented by a single lead rhythm strip, and was thus not further quantified in the majority of cases. Cases of diTdP occurring in subjects with known or later identified congenital long QT syndrome were not excluded. Those associated with clear acute cardiac ischemia (which may represent a different mechanism) were excluded. Structural heart disease was not an exclusion criterion. Age, gender, culprit drug, presence of atrial fibrillation (AF), and self-reported ethnicity were collected as covariates.

### Controls

There were three control groups: (1) Drug-challenged controls: a set of 470 subjects ascertained at Vanderbilt who were challenged with intravenous ibutilide or oral antiarrhythmics (sotalol, dofetilide, or quinidine) and who did not display any QTc interval >500 msec or any increase in QT >50 msec; and (2) 649 general population control (POPRES) [19] including Northwestern European subjects and Europeans from other sub-populations. The primary analysis was subsequently supplemented with a further 4231 British subjects from the Wellcome Trust Case Control Consortium phase 2 (WTCCC2), downloaded from http://www.ebi.ac.uk/ega/page.php. Because diLQTS is rare in the population, using a large population control set increases power even recognizing the (small) potential for misclassification [20]. Population controls have been successfully used in this fashion in previous studies of rare adverse drug reactions in which GWAS did yield significant associations [16], [21].

### Ethics statement

Cases recruited at Vanderbilt Medical Center provided written informed consent for participation under a protocol entitled “Screening for Genetic Causes of Cardiac Arrhythmias” approved by the Vanderbilt Human Research Protection Program (date of latest approval Dec. 11, 2012). All patients give their written consent to participate, and the signed consent form is retained in the patients’ research chart to document the consent process. For minors, written consent was obtained from parent/guardian. For minors ages 12 and older, written consent was obtained from the child also. Under the age of 12, verbal assent was obtained from the child. The signed consent form is retained in the patients’ research chart to document the consent process.

Drug-exposed controls provided written consent under a protocol entitled “Pharmacogenomics of Arrhythmia Therapy-Ion Channel Variants and QT-Prolonging Antiarrhythmics” approved by the Vanderbilt Human Research Protection Program (date of latest approval Dec. 13, 2012). All patients give their written consent to participate, and the signed consent form is retained in the patients’ research chart to document the consent process. For minors, written consent was obtained from parent/guardian. For minors ages 12 and older, written consent was obtained from the child also. Under the age of 12, verbal assent was obtained from the child. The signed consent form is retained in the patients’ research chart to document the consent process. Cases accrued by the Leducq network provided written consent approved by local ethics boards. These are: at Nantes: CPP and ministère de la recherche: biocollection de l’institut de thorax intitulée « Génétique et biomarqueurs des pathologies cardiovasculaires, respiratoires et leur facteur de risques ». Declared under the ref n° DC-2011-1399; at Münster: local ethics committee, University Hospital Münster; at Amsterdam: Medisch Ethische Commissie AMC (Medical Ethical Committee Academic Medical Centre, University of Amsterdam, Amsterdam); at Munich: Ethikkommission bei der LMU München; at Miami: University of Miami Institutional Review Boards. Consent for inclusion in the DARE study was given under Multi-Centre Research Ethics Committee approval MREC 02/02/73 on February 24th 2003 with minor amendment approval given on February 3rd 2010.

### Genotyping

The Leducq cases and the drug-exposed controls were genotyped on the Illumina 610k platform by the RIKEN Center for Genomic Medicine (Yokohama, Japan) as part of a collaboration between RIKEN and the National Institutes of Health-supported Pharmacogenetics Research Network. The DARE cases and the POPRES and WTCCC2 controls were genotyped using the Illumina 1M or 1M-Duo chip. We used a consistent QC procedure for each genotyping assay, as previously described [16], [21]: SNPs required a call rate > 0.95, minor allele frequency > 0.01, and Hardy Weinberg Equilibrium (HWE) in controls p>10^−7^, and subjects required a call rate > 0.9. We tested for cryptic relatedness by estimating identity-by-descent (IBD) for all possible pairs of individuals using PLINK [22].

### Principal components analysis (PCA)

We used the smartPCA program from the EIGENSTRAT package (version 3.0) [23] to conduct a PCA in order to expose population structure. SNPs from known regions of long-range linkage disequilibrium [24] were removed before conducting the PCA. We first analyzed the study genotype data to identify major ancestry groups (Europeans, Asians and Africans). We then conducted a PCA on Europeans to separate sub-populations within this group.

### Statistical analyses

We conducted statistical tests using PLINK v1.05 [22]. The primary analysis compared the cases of Northwestern European ancestry to genetically matched controls. We conducted analyses comparing cases to drug-exposed controls, to the POPRES population controls, to drug-exposed + POPRES controls, and to drug-exposed + POPRES + WTCCC2 controls. The initial analysis was conducted on the set of SNPs common across all sets, and as discussed below, we also extended the analysis to an imputed set. We also analyzed drug-specific sets for the three most common culprit agents, sotalol, amiodarone and quinidine, and searched for associations using gene-specific analyses, as described further below.

**Figure 1:**
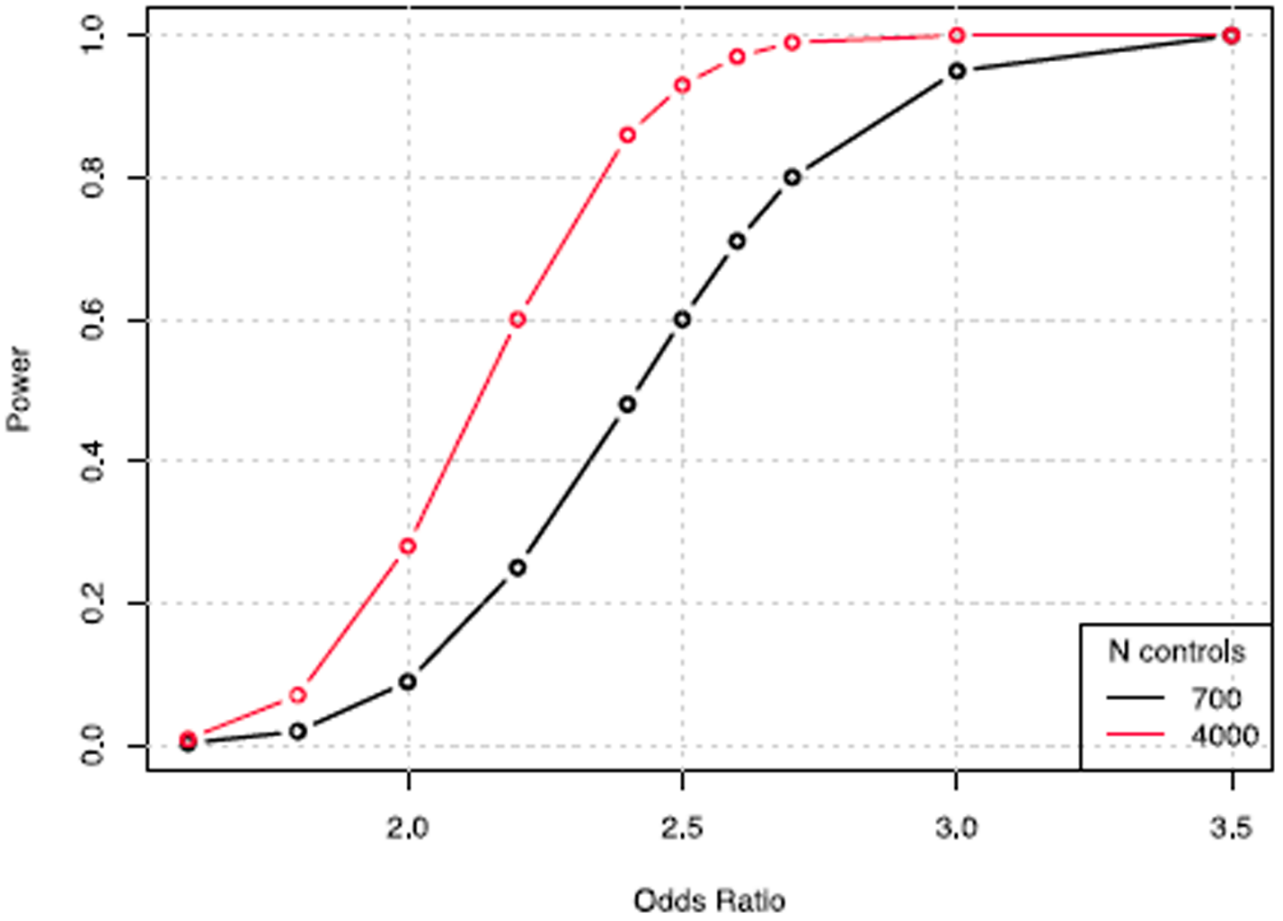
Plot of power versus odds ratios, using 700 controls (initial analysis) and then >4000 controls.

We tested the association of single SNPs with diTdP susceptibility using logistic regression, with gender and Eigen score as covariates in an additive model. We set the genome-wide significance threshold of P-value at to 5×10^−8^ to correct for multiple testing. The power calculation with Bonferroni correction was based upon the following assumptions: 265 cases; a low prevalence of diTdP in the general population; a SNP minor allele frequency (MAF) of 0.1; a 0.8 R^2^ coefficient between the SNP and the causal allele; and an odds ratio (OR) in the range of 1.5 and 2.5. The power was calculated based on a chi-square test under an additive model, using a non-centrality parameter [25]. This supported the use of the WTCCC2 controls to achieve increased power (to 29% for OR  =  2.0, to 93% for OR  =  2.5, and to 99% for OR  =  2.7), in the subsequently analysis of the selective cohorts (Figure 1).

We calculated r^2^ among top associated SNPs located in a 1M bp interval. In the case of two or more SNPs not correlated with each other, we conducted a haplotype analysis using PLINK.

### Imputation

Imputation of missing genotypes was carried out using IMPUTE2 (February 2009 version) [26], with data from the 1000 Genomes Project (56 individuals, August 2009 release) and HapMap III (146 individuals, CEU and TSI) as the reference panels. All known SNPs with poor quality were removed before the imputation to avoid false positives. Imputed genotypes with a posterior probability of greater than 0.9 were retained [26]. To assess the accuracy of the imputation results, we carried out the following quality control procedures: (1) we tested the difference of missingness between cases and controls; (2) we tested for HWE (p-value << 0.05); 3) we manually inspected signal quality for all the SNPs in the haplotype generating the imputed genotype.

### Gene-based approaches

We also performed a gene-based GWAS analysis. In this analysis we aggregated SNPs by the genes they are near to increase the signal. This approach is based on the idea that multiple variants in a single gene will perturb gene function [27]. For each patient in the cases and the controls, we scored each gene by counting the number of minor alleles in that gene. These counts are weighted by the negative log of the frequency that the allele is observed at in the population. This approach up-weights rare alleles and down-weights common alleles [27]. We then performed a univariate logistic regression modeling the cases versus controls as a function of these gene scores. We performed an F-test to assess the significance of the fit.

### Repository information

Portions of the Leducq dataset approved by local IRBs for central deposit have been uploaded to dbGaP at http://www.ncbi.nlm.nih.gov/projects/gap/cgi-bin/study.cgi?study_id=phs000331.v1.p1. The DARE dataset is held by the International Serious Adverse Events Consortium (iSAEC) and is available on application to the iSAEC data management committee at http://www.saeconsortium.org/.

## Results

There was a total of 264 cases genotyped, 210 from the Leducq network and 54 from the DARE study. The clinical features of these sets are presented in Table 1. They included 185 women (71%), the age range was 7–91, and the most commonly-implicated drugs were sotalol, amiodarone and quinidine (Table 2). The majority self-declared European ancestry (98% for the DARE study and 92% for the Leducq network).

**Table 1:** diTdP Genome-Wide Association Study: Demographic and clinical characteristics of the enrolled cases summarized by cohort.

Cohort	Number of cases enrolled	Number of North-western European cases	Age at the onset (Mean, SD)	Female (% of total)	Atrial Fibrillation (Yes, % of total)	% self-defined Europeans
Leducq	210	163	61.9±16.5	71%	49%	92% *
DARE	54	53	66.8±15.9	64%	52%	98%
Total	264	216	62.5±16.3	70%	50%	

* In total, 66% of the Leducq cases self-declared ethnicity, among then 92% (61% of total) are Europeans.

**Table 2:** diTdP Northwestern European cohort: causal drugs and demographic and clinical characteristics of the diTdP Northwestern European cases summarized by drug group.

Drugs	# Cases	Atrial Fibrillation (Yes, %of total)	Age (Mean, SD)	% Female
Amiodarone	46	50%	62.5±17.1	65%
Quinidine	19	63%	55.6±22.3	68%
Sotalol	55	53%	55±26.5	75%
Others	141	36%	58.2±23.8	72%

Subjects might have experienced diTdP due to more than one causal drug.

### Quality control and population structure

Genotype data from the 264 cases and 1119 drug-exposed and POPRES controls underwent QC as described above. After dropping SNPs with genotyping efficiency ≤95% or MAF ≤1%, the final set for initial analysis across all platforms used included 478,055 SNPs. Of the 1383 samples, 46 sample pairs had identity-by-descent sharing values > 0.2 in the Leducq set, and we removed the sample from each pair with the lower overall SNP calling rate. In total, we selected for further analyses 242 cases from all ethnicities (189 from the Leducq set and 53 from the DARE set) and 1094 controls (435 drug-exposed control and 659 POPRES controls).

**Figure 2:**
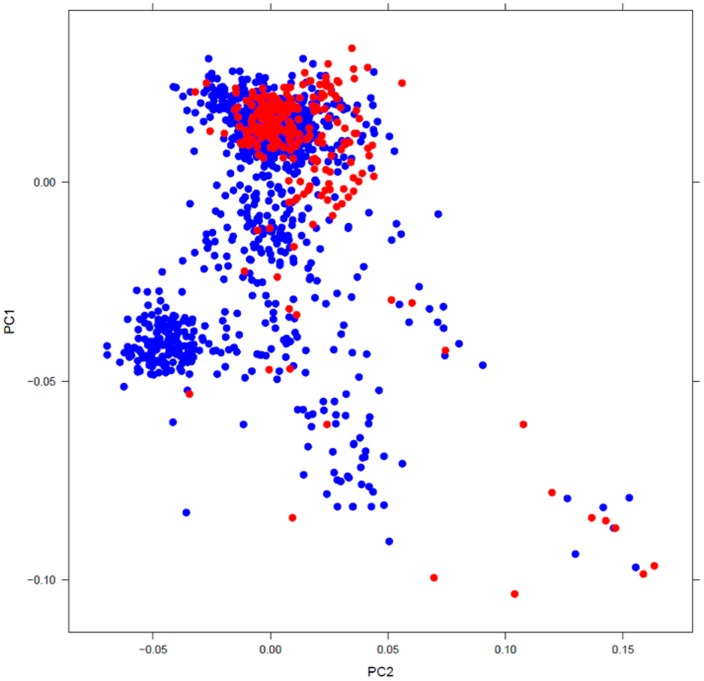
Population structure of the Caucasian cases and controls. The red dots represent diTdP cases and blue dots represent controls (drug-exposed patients and population [POPRES] controls). The plot shows the first and second eigen vectors, which clearly separate the Caucasians into a Northwestern group (top) and other groups from Southern and Eastern Europe. The dense cluster on the lower left represents the subjects of Spanish origin from the POPRES collection. The final analysis included subjects with PC1<–0.03.

After identifying and removing non-Caucasian subjects by PCA, we investigated the genetic structure of Caucasian cases and drug-exposed controls. As shown in Figure 2, most cases and controls clustered in the Northwestern European cohort. We therefore used samples with PC1>-0.03 to make up the final sets for the primary analysis; this included 216 cases, 386 drug-exposed controls, and 385 POPRES controls.

### Genome-wide statistical analysis

**Figure 3:**
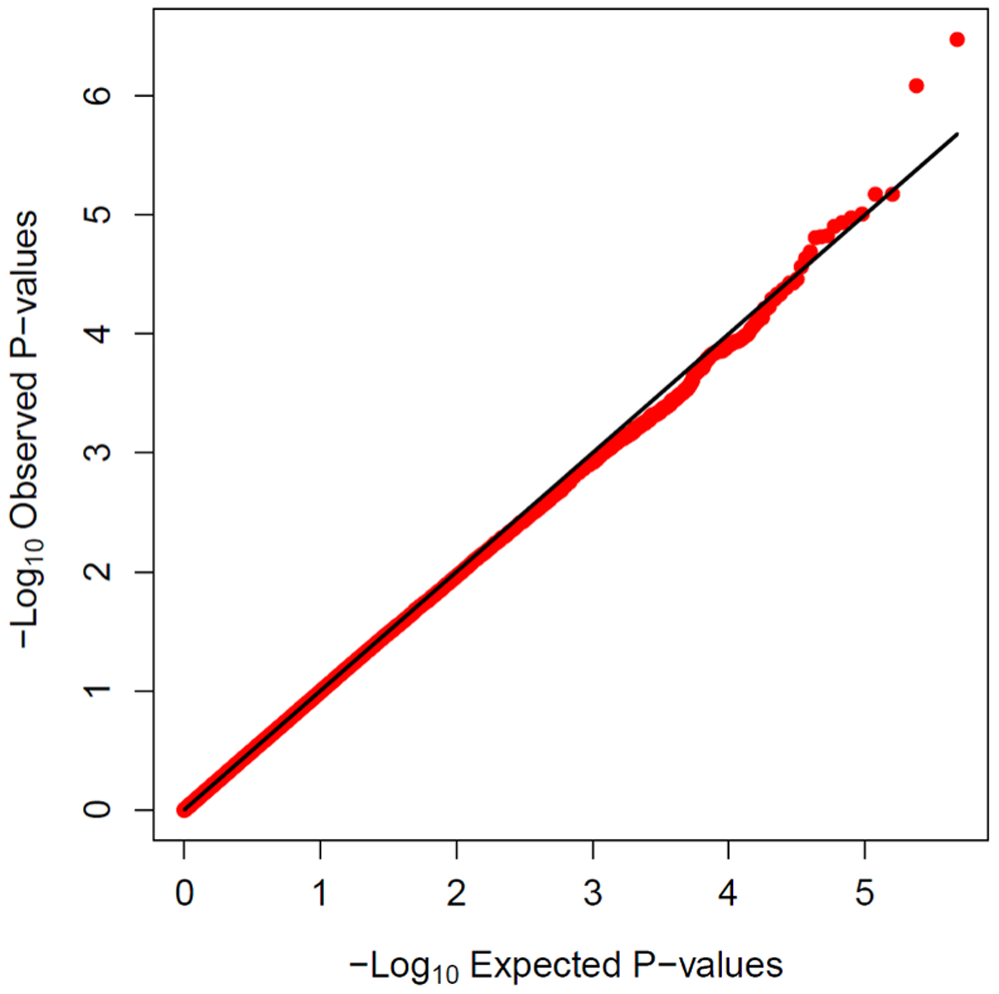
QQ plot of the results from logistic regression on the Northwestern European cohort. The x axis is –log10 of the expected P-value and the y axis is –log10 of the observed P-values. Black solid lines denote the null distribution. The bulk of the values (red dots) closely follow the expectation under the null model (black line) showing that there is no significant inflation of test statistic due to factors such as population stratification. The tail end shows significant deviation from null model illustrating that there are a few observed significant associations.

**Figure 4:**
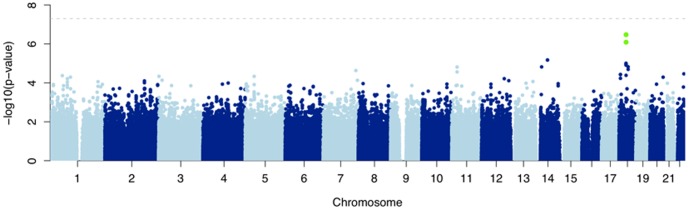
Manhattan Plot of logistic regression on Northwestern European cohort. Each dot represents a SNP. The x axis represents the position of the SNP on chromosome. The y axis represents the -log_10_ of logistic regression P-value of the SNP in the case-control association study. rs2276314 and rs4799838 are marked in green with the P-value just below the genome-wide threshold (dashed line).

Using logistic regression with the first three significant principal components and gender as covariates (Figure 3 and 4), we found no SNPs with p-values <10^−7^ in separate analyses of cases versus drug-exposed controls, cases versus population controls, and cases versus the combined control set (Table 3). In the combined analysis (Figures 3 and 4), the SNP with the lowest P value in an additive model (rs2276314, P = 4×10^−7^, OR = 2, 95% CI 1.5–2.7) was a non-synonymous coding variant in *C18orf21*, a gene thought to be transactivated by Hepatitis B virus. There was no significant difference in MAF between the treatment-tolerant and general population groups (Table 4), or between subjects with or without AF, indicating that the association is not likely to be influenced by confounding factors. Interestingly, the haplotype AA formed by rs2276314 and rs767531, a nearby SNP, was significantly more frequent in controls (79% of controls vs. 65% of cases) with a chi-square p-value of 1.6×10^−9^. Rs767531 is an intragenic SNP close to *GALNT1,* which is thought to mediate O-linked glycosylation in the Golgi.

**Table 3:** diTdP Genome-Wide Association Study: top associated SNPs.

SNP	CHR	Position	functional type	closest gene	OR	P-value
rs2276314	18	33557466	Non synonymous	C18orf21	2.0 (1.5–2.6)	4.0×10^−07^
rs4799838	18	33597530	Intronic variant	RPRD1A	1.9 (1.5–2.5)	9.4×10^−07^
rs9960370	18	33494636	Intergenic variant	MIR187	2.0 (1.4–2.7)	6.0×10^−06^
rs767531	18	33357868	Intergenic variant	GALNT1	3.0 (1.8–4.8)	7.1×10^−06^
rs1789536	18	33524398	Intergenic variant	C18orf21	2.0 (1.4–2.7)	7.4×10^−06^
rs1789532	18	33532322	Intergenic variant	C18orf21	2.0 (1.4–2.6)	7.9×10^−06^
rs10141001	14	52132201	Intronic variant	FRMD6	2.0 (1.4–2.7)	1.0×10^−05^
rs9805984	14	52131743	Intronic variant	FRMD6	2.0 (1.5–2.7)	1.0×10^−05^

Abbreviations: OR (Odds Ratio, OR is expressed with a confidence interval of 95%), CHR (chromosome).

**Table 4:** Minor Allele Frequency (MAF) for rs2276314, the top associated SNP, in different sample sub-groups.

sub-groups	MAF
DC controls (382)	0.19
POPRES controls (389)	0.19
subjects with AF (371)	0.23
subjects without AF (182)	0.21

Abbreviations: AF (atrial fibrillation), MAF (minor allele frequency).

**Figure 5:**
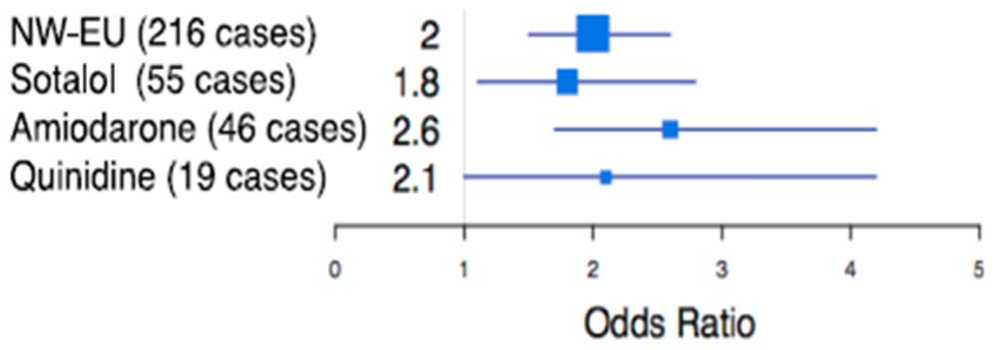
The effect size (OR) of rs2276314 in different drug-specific groups. The numbers in parentheses are the numbers of cases for each group. The horizontal blue lines mark the 95% confidence interval of odds ratios.

We investigated drug-specific risk alleles by comparing the 771 Northwestern European control subjects with three drug-specific Northwestern European case groups (Figure 5): sotalol (55 cases), amiodarone (46 cases), and quinidine (19 cases). We found no SNP with genome-wide significance in any of the three comparisons.

An initial analysis of the two cohorts separately did suggest possible signals at or near genome-wide significance, but these signals were not observed in the combined analysis presented here. For example, the initial analysis of the DARE set identified rs2200733 as a potential risk allele. This variant has previously been associated with AF [28] and thus may represent the fact that antiarrhythmic drugs, the most common culprit agents for diTdP, are most often prescribed for patients with AF. The association was not seen in the Leducq set or in the combined analysis.

### Alternate analytic approaches

We used imputation to increase the number of SNPs evaluated to 3,542,142 as described in the methods section. We repeated the phenotype and drug specific analyses and we found no genome-wide significant associations. We also obtained no significant associations by including the WTCCC2 controls.

Using the gene-based GWAS analysis method described, we did not find any significant associations at the p<10^−6^. The most significant association encoded for a cytochrome P450 enzyme, CYP2U1 on chromosome 4 (p = 1.2×10^−5^), followed by ubiquitin, USP25 on chromosome 21 (p = 3.4×10^−5^), and a G protein-coupled receptor, OPRL1 on chromosome 20 (p = 9.2×10^−5^).

As mentioned above, small series have identified occasional patients with incompletely penetrant congenital long QT syndrome that becomes manifest with drug exposure [6]–[8]. In a minority of diTdP cases analyzed here (n = 66–132), coding region resequencing in six congenital long QT syndrome disease genes had previously been undertaken. As shown in Table 5, this effort identified 10 rare variants implicated as function-altering likely congenital syndrome mutations [6], [29], [30].

**Table 5:** Screening rare variants in congenital long QT Syndrome disease genes in 216 diTdP cases.

	*KCNQ1*	*KCNH2*	*SCN5A*	*ANK2*	*KCNE1*	*KCNE2*
Number screened	131	132	129	66	89	80
Rare variants identified	3	3	1	2	1	0
Rare variants identified	W305X	R784W	A385T	T1404I	D76N	
	T224M	G934V		V1516D		
		R954C				

## Discussion

Severe adverse events during drug therapy are by their nature unpredictable: if markers of high risk are known, they could be used to reduce or even eliminate risk in an individual subject or for a drug or drug class. The very unpredictable nature of many such events has prompted a search for underlying markers of genomic susceptibility. In the case of diTdP, a contribution by a genomic component is especially appealing given the clinical similarity to the congenital long QT syndrome, evidence that QT interval responses to drug challenge may be exaggerated among relatives of those with diTdP, and the known heritability of QT duration.

One of the major impediments to identifying genomic markers of risk for TdP or other severe adverse drug reaction is the very rarity of the event that precludes accumulation of large numbers of cases [31]. This integrated analysis of two large multi-institutional ascertainment efforts presented here represents the largest such effort for diTdP to date. The analysis did not identify common variants conferring high risk for diTdP. This negative finding suggests that it is unlikely that any single common genomic markers of risk with odds ratios greater than 2.5 contribute. The result leaves open the possibility that predisposition to diTdP is determined by interactions of multiple variants each with modest effect sizes, or that rare variants play a prominent role. Indeed, a recent large-scale candidate gene analysis of the Leducq set implicated an uncommon non-synonymous variant in the potassium channel subunit *KCNE1*: minor allele frequency in control populations was 1.8–2.9% and 8.6% in cases [32]. A trend in the same direction was seen in the DARE set.

In addition, environmental factors may also be important; these include electrolyte abnormalities, alterations in autonomic tone or heart rate, or underlying heart disease. Indeed, the signal observed in the DARE set between AF risk alleles and diTdP may represent confounding by indication (i.e. AF is an indication for the culprit drugs) or may represent an association between homeostatic perturbations induced by AF and subsequent susceptibility to diTdP. Studies of QT interval during AF have identified abnormal control of repolarization (very flat QT-RR relationships; i.e. absence of expected QT prolongation at slower heart rates) during the arrhythmia, with normalization or even super-normalization (i.e. longer than expected QT interval at any given heart rate) after conversion to normal rhythm [33], [34]. The mechanism underlying these changes has not been elucidated, although it is known that the neurohormonal milieu does change shortly after conversion to normal rhythm: for example, catecholamines [35] and atrial natriuretic peptides [36] are higher during AF than after conversion. These changes may contribute to altered QT-RR regulation and susceptibility to diTdP after conversion to normal rhythm [33].

The SNP with the lowest P value is a non-synonymous variant in *C18orf21* that results in a substitution of an alanine for a threonine at position 132 of the protein. The threonine is a predicted phosphorylation site (according to the NetPhos 2.0 website). The function of this transcript is unknown, so this signal may represent new biology or a false positive.

### Limitations

In contrast to the present study, the GWAS paradigm has been used to identify single loci with strong effects in other adverse drug reactions. Carbamazepine-related skin toxicity [17] and flucloxacillin-related hepatotoxicity [16] were linked to variants in the HLA locus in studies using discovery sets of 65 and 51 cases respectively. Similarly, a GWAS of 85 cases of myotoxicity related to high dose simvastatin identified variants at the *SLCO1B1* locus, encoding a statin drug transporter [18]. The present study also examined a rare adverse drug reaction and included 216 cases with a well-defined genetic background, but did not identify loci strongly associated with diTdP. We believe this indicates that variants at a single locus do not have a strong influence on diTdP risk across drugs and drug classes. Basic electrophysiologic studies have implicated a common mechanism across multiple drugs, block of the rapid component of the delayed rectifier potassium current I_Kr_, in diTdP [4], [37] and justify the inclusion of multiple culprit drugs in the present study. This design will not detect drug-specific mechanisms predisposing to diTdP; examples could include alterations in elimination pathways or in drug-specific (e.g. non-I_Kr_-mediated) electrophysiologic pathways leading to diTdP [38]. The analysis by drug performed here did not yield any suggestive signals, but the numbers are small; similarly, an analysis by gender did not yield significant associations. We also did not exclude patients with heart failure, which is associated with QT prolongation. A recent study of amiodarone-related diTdP [39], using the multiple SNPs interrogated by the cardiochip, has suggested that variants in the *NOS1AP* locus, known to modulate baseline QT interval [14], [15], also confer risk for amiodarone-related TdP. Rare variants may contribute to susceptibility, although screening in congenital long QT syndrome disease genes yielded likely contributory mutations in 0–3% (depending on the gene; Table 5) of subjects.

Small numbers of cases represent a generic problem in the study of rare adverse drug events and support the development of further multi-institutional efforts to accrue cases and controls under uniform protocols and definitions [31], [40]._ENREF_24 The present study cannot address whether there are common genomic variants that confer risk in subjects of non-Northwestern European ancestry. One small association study has suggested that the common S1103Y variant in the cardiac sodium channel gene *SCN5A*, seen almost exclusively in subjects of African origin, confers increased risk for a range of arrhythmias, including diTdP [41]. Follow-up clinical and *in vitro* studies have further supported a role for this variant in arrhythmia susceptibility [42], [43]. It is possible that gene-gene interactions drive susceptibility to diTdP but the present study is not powered to detect such associations.

In conclusion, the results described here represent analysis of the largest number of genotyped cases of diTdP reported to date, and effectively rule out any likely role for common variation with anything other than modest effects. Further efforts to define risk for the adverse drug event would benefit from accrual of larger patient sets and extension of analytical approaches to sequence-based analysis to further explore the hypothesis that rare variants (e.g. in congenital arrhythmia syndrome genes) mediate diTdP risk. The precise role of genetic variation in the risk of diTdP remains to be determined.
